# Combined Gemcitabine and Immune-Checkpoint Inhibition Conquers Anti-PD-L1 Resistance in Low-Immunogenic Mismatch Repair-Deficient Tumors

**DOI:** 10.3390/ijms22115990

**Published:** 2021-06-01

**Authors:** Inken Salewski, Julia Henne, Leonie Engster, Bjoern Schneider, Heiko Lemcke, Anna Skorska, Peggy Berlin, Larissa Henze, Christian Junghanss, Claudia Maletzki

**Affiliations:** 1 Department of Medicine, Clinic III–Hematology, Oncology, Palliative Medicine, Rostock University Medical Center, University of Rostock, 18057 Rostock, Germany; Inken.salewski@med.uni-rostock.de (I.S.); julia.henne@uni-rostock.de (J.H.); leonie.engster@uni-rostock.de (L.E.); Larissa.henze@med.uni-rostock.de (L.H.); Christian.junghanss@med.uni-rostock.de (C.J.); 2 Institute of Pathology, Rostock University Medical Center, University of Rostock, 18057 Rostock, Germany; bjoern.schneider@med.uni-rostock.de; 3 Department of Cardiac Surgery, Reference and Translation Center for Cardiac Stem Cell Therapy (RTC), Rostock University Medical Center, University of Rostock, 18057 Rostock, Germany; Heiko.Lemcke@med.uni-rostock.de (H.L.); anna.skorska@med.uni-rostock.de (A.S.); 4 Faculty of Interdisciplinary Research, Department Life, Light & Matter, University Rostock, 18057 Rostock, Germany; 5 Division of Gastroenterology and Endocrinology, Department of Medicine II, Rostock University Medical Center, University of Rostock, 18057 Rostock, Germany; peggy.berlin@med.uni-rostock.de

**Keywords:** immune checkpoint inhibitor, MMR deficiency, in vivo imaging, tumor microenvironment, genetic model, coding microsatellite mutations

## Abstract

Tumors arising in the context of Lynch Syndrome or constitutional mismatch repair deficiency are hypermutated and have a good response towards immune-checkpoint inhibitors (ICIs), including α-PD-L1 antibodies. However, in most cases, resistance mechanisms evolve. To improve outcomes and prevent resistance development, combination approaches are warranted. Herein, we applied a combined regimen with an α-PD-L1 antibody and gemcitabine in a preclinical tumor model to activate endogenous antitumor immune responses. Mlh1^−/−^ mice with established gastrointestinal tumors received the α-PD-L1 antibody (clone 6E11; 2.5 mg/kg bw, i.v., q2wx3) and gemcitabine (100 mg/kg bw, i.p., q4wx3) in mono- or combination therapy. Survival and tumor growth were recorded. Immunological changes in the blood were routinely examined via multi-color flow cytometry and complemented by ex vivo frameshift mutation analysis to identify alterations in Mlh1^−/−^-tumor-associated target genes. The combined therapy of α-PD-L1 and gemcitabine prolonged median overall survival of Mlh1^−/−^ mice from four weeks in the untreated control group to 12 weeks, accompanied by therapy-induced tumor growth inhibition, as measured by ^[18F]^-FDG PET/CT. Plasma cytokine levels of IL13, TNFα, and MIP1β were increased and also higher than in mice receiving either monotherapy. Circulating splenic and intratumoral myeloid-derived suppressor cells (MDSCs), as well as M2 macrophages, were markedly reduced. Besides, residual tumor specimens from combi-treated mice had increased numbers of infiltrating cytotoxic T-cells. Frameshift mutations in *APC*, *Tmem60*, and *Casc3* were no longer detectable upon treatment, likely because of the successful eradication of single mutated cell clones. By contrast, novel mutations appeared. Collectively, we herein confirm the safe application of combined chemo-immunotherapy by long-term tumor growth control to prevent the development of resistance mechanisms.

## 1. Introduction

Conventional oncological treatment regimens include surgery, radiation, and chemotherapy. While the latter is a widely applied treatment option for primary and metastatic diseases, the side effects are complex, including myelosuppression. Cancer immunotherapy is a safe and effective treatment option, and immune-checkpoint inhibitors (ICIs) are widely used nowadays, both in research and clinically to force tumor cell killing via reactivation of exhausted T-cells. Additional to the already established α-PD1 and α-CTLA-4 antibodies, the FDA recently approved atezolizumab, avelumab, and durvalumab as antibodies against PD-L1, because of proven long-lasting immune-responses in certain patient cohorts [1,2,3]. 

Lynch Syndrome and constitutional mismatch repair deficiency (dMMR) are two hereditary cancer syndromes with a high likelihood of having a good response towards ICIs. In both syndromes, germline mutations in one of the mismatch-repair genes constitute the oncogenic driver, resulting in early-onset tumorigenesis [4,5,6,7,8,9,10,11]. Lynch Syndrome carriers frequently harbor *MLH1* or *MSH2* germline mutations, whereas, in constitutional mismatch repair deficiency, germline *PMS2* and *MSH6* mutations dominate. A hallmark of all dMMR-driven tumors is the high tumor mutational burden, often characterized by frameshift mutations in coding microsatellites (cMS) of tumor suppressor genes. This hyper- or ultra-hypermutated phenotype directly correlates with the level of immunity and contributes to the approval of the α-PD-1 antibody pembrolizumab for 1st-line treatment of patients with unresectable or metastatic dMMR colorectal cancer (CRC) [12,13]. The SAMCO-PRODIGE 54 randomized phase II trial is currently evaluating avelumab vs. standard 2nd-line treatment chemotherapy in metastatic dMMR CRC patients [14]. Additional studies are ongoing, including 1st and 2nd-line treatment schedules (clinical trials.gov). 

While the enthusiasm of ICIs is often thwarted by resistance mechanisms and relapse upon successful ICI-tailored therapy, combination therapies are actively tested. A very promising approach is the combination of chemotherapeutics, based on the observation that some drugs activate endogenous antitumor immune responses [15]. These include direct effects such as the induction of immunogenic cell death, but also indirect effects via cytotoxic T cell activation and tumor infiltration. These encouraging results have contributed to the initiation of clinical trials for α-PD-L1-based chemo-immunotherapy to treat solid tumors (NCT03572400, NCT03324282, NCT03093922). Furthermore, even tumors with low mutational load (Lynch syndrome subtype G2) acquire a higher mutational burden by chemotherapy [16,17]. Gemcitabine is among the most promising drugs. Acting like a classical cytotoxic drug by inhibiting DNA synthesis, this substance has the capacity to activate the immune system and shift the tumor microenvironment towards an inflammatory milieu [18,19]. Indeed, in our previous study on Mlh1^−/−^ mice, this drug, in conjunction with a whole tumor vaccine, prolonged survival via immune modulation [20]. To move on, we combined gemcitabine chemotherapy with an α-PD-L1 ICI and analyzed the outcome.

## 2. Results

### 2.1. In Vitro Analysis

**Figure 1:**
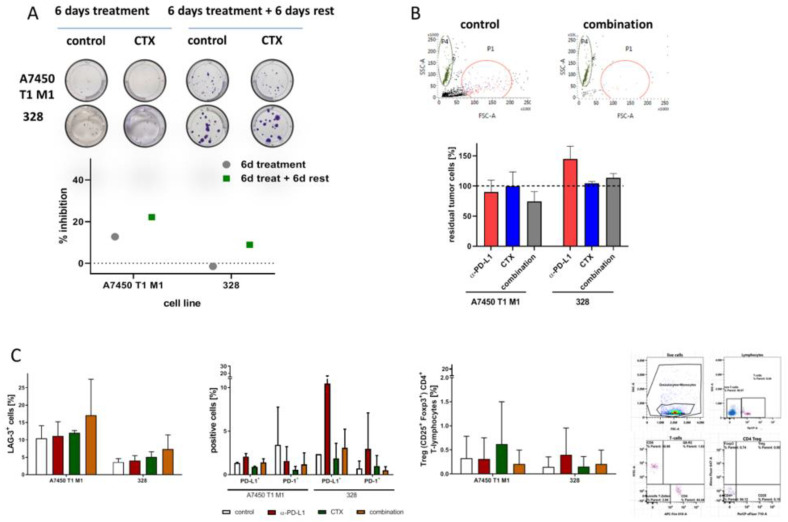
In vitro colony formation assay and co-culture. (**A**) Colony formation assay. Mlh1^−/−^ cell lines A7450 T1 M1 and 328 were treated for 6 days with gemcitabine. Colonies were counted either directly after treatment or after an additional 6 days of rest. (**B**) Co-culture of tumor cells with murine immune cells. After 72 h, cells were counted and (**C**) immune cells were phenotyped. The gating strategy is shown on the right side, next to the bar charts. Events shown within the dot plots represent mean of the numbers of positive cells ± SD resulting from 20,000 events.

Two cell lines, A7450 T1 M1 and 328, established from mouse duodenal tumors were used for preliminary in vitro experiments. The former cell line, A7450 T1 M1, was generated upon in vivo expansion, whereas 328 cells could be established from the primary tumor. Both cell lines are highly heterogenic in terms of morphology, growth kinetics, mutational profile, and drug response [21,22]. To test the efficacy of ultra-low-dose chemotherapy (CTX) treatment, a colony formation assay was performed. Figure 1A shows representative crystal violet stainings.

Experiments revealed individual responses, with more evident growth inhibition in A7450 T1 M1 cells than in 328 cells (Figure 1A). For the former, cell density was approximately 20% lower after low-dose CTX treatment and remained decreased even after 6 days of rest. For the 328 cells, a decelerated response profile was observed with a lack of initial growth inhibition, but reduced cell numbers after an additional 6 days of rest, finally reaching an inhibition of 15%. The numbers of viable cell colonies were thus below controls. Then, a semi-autologous short-term co-culture system was used to analyze the impact of the immune system. Tumor and immune cells were co-cultured in the presence of CTX, α-PD-L1, or a combination of both, and residual cells were counted after 72 h via flow cytometry (Figure 1B). Treatment with α-PD-L1 stimulated immune-mediated killing of A7450 T1 M1, but not 328 cells. CTX itself had no influence on tumor cell numbers. The combination of both agents boosted the immune-mediated killing of A7450 T1 M1 cells. Here again, 328 cells were resistant towards killing, and cell numbers even increased (Figure 1B). Then, the immune cells were stained for typical surface antigens to check for activation and exhaustion markers. Numbers of Lag3^+^ T-lymphocytes increased in the combination (Figure 1C). PD-L1^+^ and PD-1^+^ cells were lower in T-lymphocytes exposed to A7450 T1 M1 cells and the combination therapy, while both markers were elevated under α-PD-L1 treatment. No change was seen for the 328-edited lymphocytes, irrespective of the treatment. Likewise, numbers of CD4^+^CD25^+^Foxp3^+^ regulatory T cells were lower in lymphocytes co-cultured with A7450 T1 M1 cells and the combination therapy. 

Hence, though differences did not reach statistical significance, the above data hinted towards the successful elimination of tumor cells because of immune-editing. 

### 2.2. Combination of α-PD-L1 and CTX Prolongs the Survival of Mlh1^−/−^ Mice

**Figure 2:**
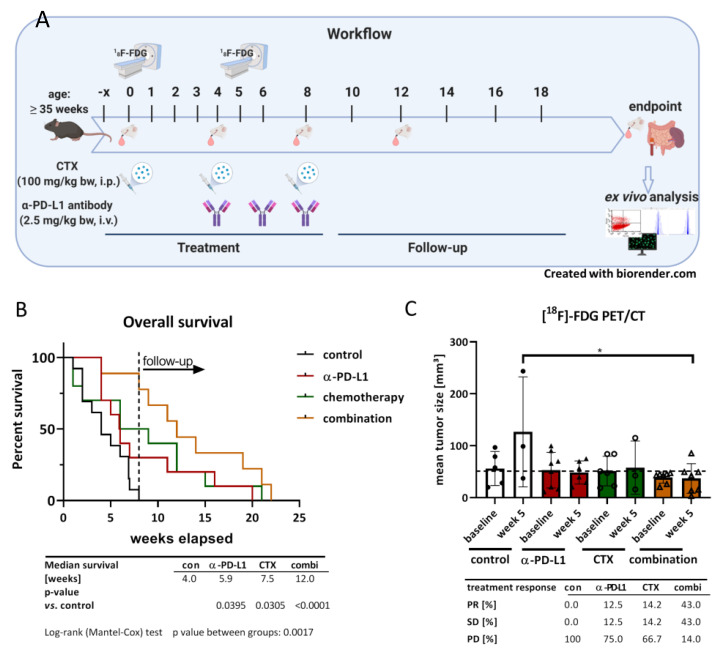
Therapy protocol and Kaplan-Meier survival curve. (**A**) Schematic therapeutic protocol including PET/CT imaging, blood collection and endpoint procedure. (**B**) Kaplan-Meier survival curve. Mice were treated with α-PD-L1 (clone 6E11, Genentech, 2.5 mg/kg bw, i.v., q2w), gemcitabine (100 mg/kg bw, i.p., q4wx3, CTX = chemotherapy), or a combination of chemotherapy, followed by α-PD-L1 antibody therapy four weeks later. (**C**) Tumor progression under therapy, measured with ^[18F]^FDG PET/CT. The mean tumor volume ± SD in mm³ are shown. Measurements were taken at start of the respective therapy and 5 weeks later (n = 3–8 mice/group and time-point). * *p* < 0.05; one-way ANOVA (Dunnett’s multiple comparison test).

Then, we tested our treatment strategy in an in vivo Mlh1^−/−^ model (see the workflow, Figure 2). Mice with an already diagnosed gastrointestinal tumor (GIT) received the α-PD-L1 antibody, CTX, a combination of both, or were left untreated (tumor size at the starting point: ≈50 mm³). Survival time of the mice was significantly influenced by the different treatments. Monotherapies with either α-PD-L1 or CTX doubled the survival rate from 4 weeks (median survival) to around 6 to 7 weeks (*p* < 0.05 vs. control). The combination of both therapies has even prolonged overall survival, reaching 12 weeks (*p* < 0.0001 vs. control, Figure 2B). Accompanying longitudinal tumor volume analysis using ^[18F]^-FDG PET/CT revealed effective tumor growth control in all three treatment groups and significantly decreased tumor size in the combination group (*p* < 0.05 vs. control, Figure 2C). Although differences were insignificant between treatment groups, we want to emphasize that all mice in the combination group received follow-up screening, whereas only 75% and 50% of mice in the α-PD-L1 and CTX group, respectively, were available for PET/CT screening. The remaining mice had to be euthanatized because of progressive disease. The combination therapy induced stable disease (SD) or partial response (PR) in 43% and 43% of mice, respectively. In the monotherapies, less than 50% of mice experienced SD or PR, and all control mice suffered from progressive disease (Figure 2C).

### 2.3. Peripheral Immune Activation by Combinational Therapy

**Figure 3:**
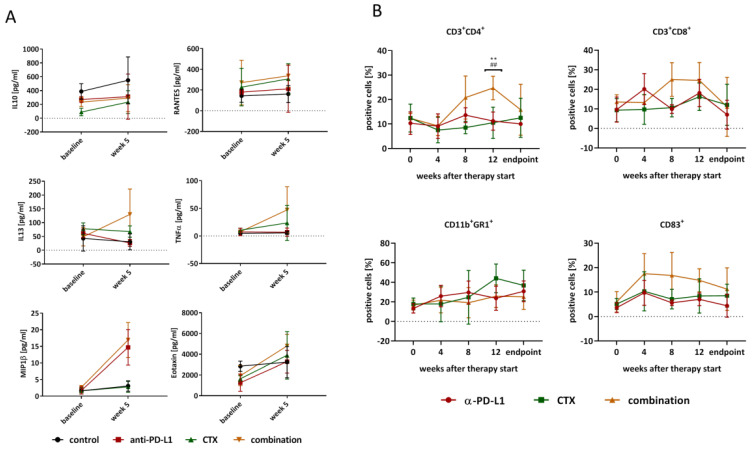
Quantification of plasma cytokine levels and assessment of immunologically relevant markers in the blood. (**A**) Cytokine levels in the plasma were analyzed at beginning of therapy and after 5 weeks. The y-axis shows the mean cytokine levels ± SD. (**B**) Every four weeks, blood was taken from mice under therapy to investigate immunological changes via flow cytometry. Represented are the numbers of positive cells ± SD resulting from 20,000 events, n = 3–5 mice/group. ** *p* < 0.01 vs. CTX; one-way ANOVA (Bonferroni’s multiple comparison test); ## *p* < 0.01 vs. α-PD-L1 monotherapy one-way ANOVA (Dunnett’s multiple comparison test).

Furthermore, immunological changes in the blood were recorded routinely via flow cytometry (Figure 3). The plasma was analyzed using a multiplex cytokine assay (Figure 3A). IL10 and RANTES concentrations did not change over time in any of the four groups, whereas, in the combination, IL13, TNFα, MIP1β, and EOTAXIN levels increased, which might be due to an increase in cytokine-secreting T cells. Besides, α-PD-L1 monotherapy also increased MIP1β concentration to the same level as the combination treatment. Additional flow cytometric phenotyping of blood samples showed quite similar levels of positive cells in both monotherapy groups, while the combination therapy induced CD3^+^CD4^+^ T-helper cells significantly after 12 weeks of treatment (*p* < 0.01 combination vs. α-PD-L1; *p* < 0.01 combination vs. CTX, Figure 3B). Moreover, the CD3^+^CD8^+^ cytotoxic T cells increased by trend in the combination group as well as CD83^+^ cells, indicative of activated B-cells and dendritic cells. The numbers of CD11b^+^GR1^+^ myeloid-derived suppressor cells (MDSCs) did not differ between individual treatment groups. 

### 2.4. Spleens and Residual Tumors Change Their Immunological Profile

**Figure 4:**
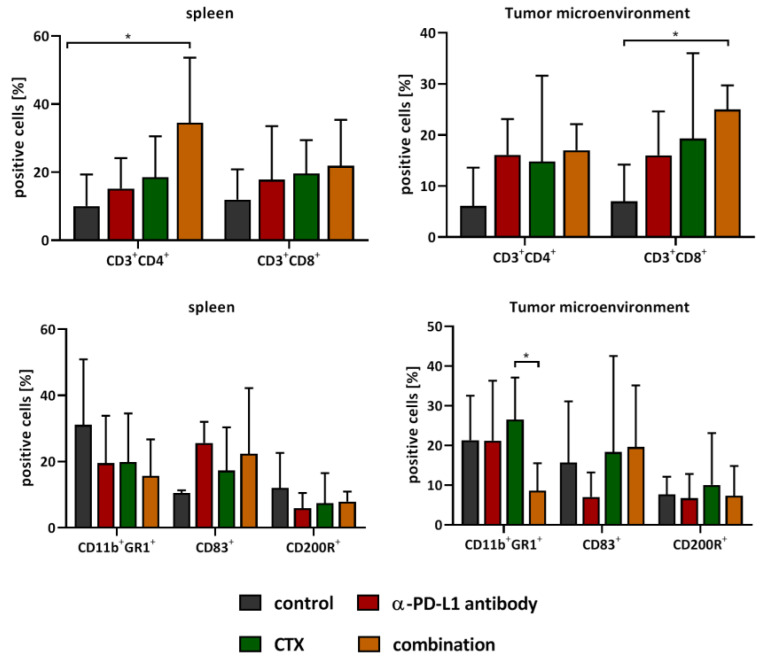
Flow cytometric phenotyping of spleens and tumor microenvironment. At the endpoint, spleen and tumor were resected. The single cell suspension was stained with respective antibodies and screened for immunological markers via flow cytometry. Represented are the numbers of positive cells ± SD resulting from 20,000 events, n = 3–5 mice/group. * *p* < 0.05 one-way ANOVA (Bonferroni’s multiple comparison test).

At the endpoint, either determined by humane endpoints or after several weeks of follow-up observation, spleens and residual tumors were analyzed using flow cytometry (Figure 4). Similar to the blood, numbers of CD3^+^CD4^+^ T-helper cells significantly increased in the spleen after combinational treatment (*p* < 0.05 vs. control). CD3^+^CD8^+^ cytotoxic T cells and CD83^+^ cells were slightly increased after all three therapies. In contrast, CD11b^+^GR1^+^ MDSCs and CD200R^+^ cells decreased upon treatment. The therapy effect on tumor infiltrating T cells goes hand in hand with the effect in the spleen. All three groups were characterized by increased T cell levels, reaching significance in the combination (CD3^+^CD8^+^ T cells, *p* < 0.05 vs. control). The percentage of CD11b^+^GR1^+^ MDSCs was as high as in the control in the monotherapies but decreased significantly after combination therapy. The numbers of CD83^+^ cells did not change considerably but declined in the α-PD-L1 monotherapy. No effect was seen for CD200R^+^ cells, implicating a minor role in the immune regulatory functions of CD200-CD200R interaction.

**Figure 5:**
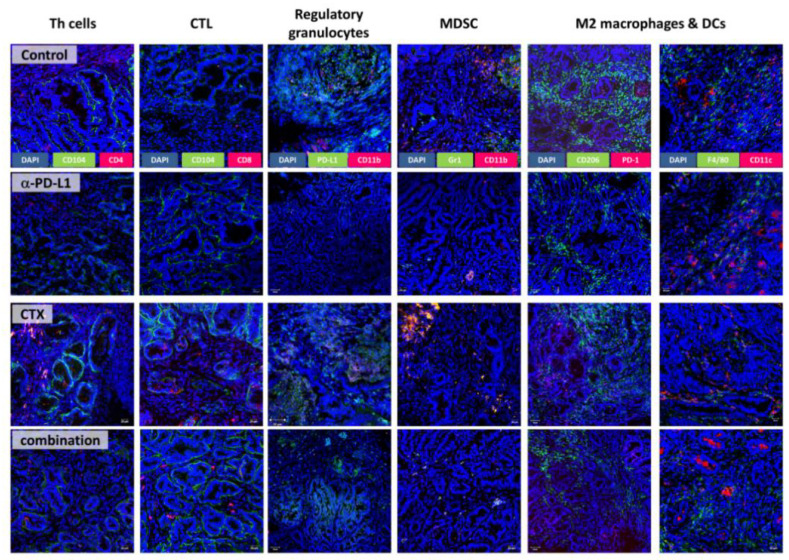
Immunofluorescence. Mlh1^−/−^ GIT cryostat sections of 4 µm were prepared and the tumor microenvironment was studied upon staining with specific monoclonal antibodies, followed by nuclear staining with DAPI. Images were taken using a laser scanning microscope, Elyra PS.1 (Zeiss), and 20× objective.

**Figure 6:**
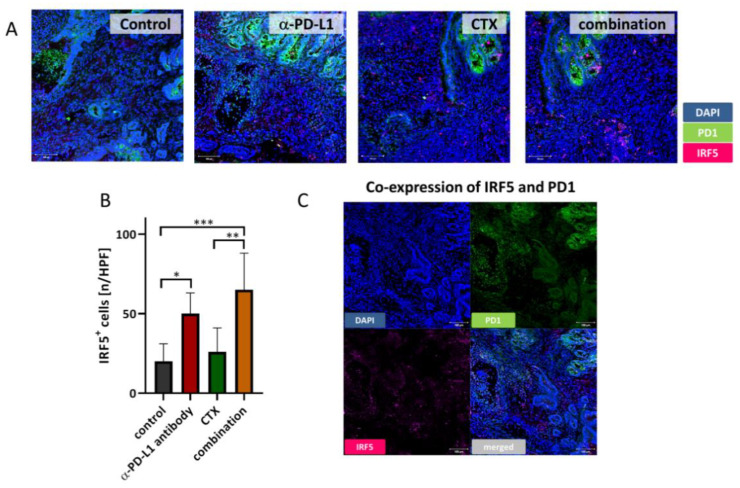
IRF5 immunofluorescence. (**A**) Mlh1^−/−^ GIT cryostat sections of 4 µm were prepared and stained with Alexa488-conjugated anti-PD1 antibody and primary rabbit anti-IRF5 antibody, followed by staining with a secondary Alexa647-labeled antibody. Nuclei were counterstained with DAPI. Pictures were taken on a laser scanning microscope (Zeiss) using 20× objectives. (**B**) Quantification of IRF5^+^ infiltrating immune cells. At least three images were taken from each slide and numbers of infiltrating cells counted. Data are given as infiltrates/high power field (HPF). Mean + SD, n ≥ 3 samples/group; * *p* < 0.05; ** *p* < 0.01; *** *p* < 0.001; one-way ANOVA (Bonferroni’s multiple comparison test). (**C**) Representative immunofluorescence images show co-localization of IRF5^+^ cells and PD1^+^ cells (single planes and merged channels).

In addition to flow cytometry, the tumor microenvironment was studied in detail by fluorescence microscopy (Figure 5 and Figure 6). The amount of cytotoxic T-lymphocytes was higher upon CTX and the combination treatment. T-helper cells additionally increased by CTX. MDSCs were only visible in the control and CTX groups, and thus effectively eliminated by the α-PD-L1 antibody. M2 macrophages and DCs were present in all groups, but their density and location differed between groups (Figure 5). PD-1 expressing M2 macrophages vanished in the combination, in a similar manner to regulatory granulocytes, which were more abundant in the control and CTX groups. Hence, the α-PD-L1 antibody itself shaped the tumor microenvironment by eradicating immunosuppressive cell populations (i.e., CD11b^+^PD-L1^+^, CD206^+^PD1^+^) and promoting infiltration of antigen-presenting cells (CD11c^+^). In support of this, we also found significantly higher levels of IRF5^+^ cells within tumor sections treated with the α-PD-L1 antibody alone or in combination with CTX (Figure 6A,B). In most cases, IRF5^+^ cells co-localized with PD1^+^ cells in the lymphoid compartment within the tumors (Figure 6C). Hence, IRF5 may constitute a direct indicator for successful immune activation in α-PD-L1-based regimens.

### 2.5. Treatment with α-PD-L1 Induced Molecular Changes in cMS of dMMR-Target Genes

**Figure 7:**
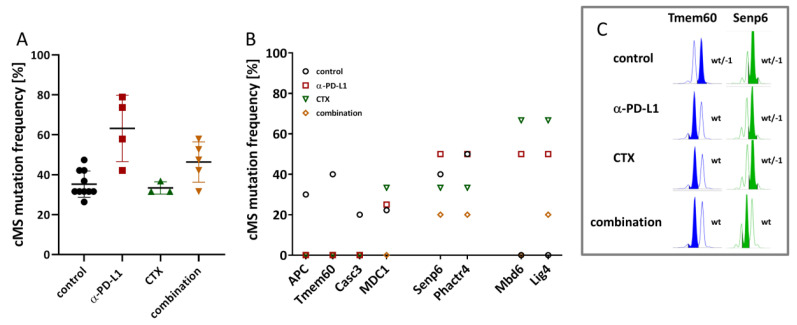
cMS mutation frequency. (**A**) Fragment length analysis was used to detect frameshift mutations in selected cMS. Overall mutational frequency in residual tumors revealed higher mutational load after α-PD-L1 monotherapy and the combination. (**B**) The cMS mutation frequency in selected Mlh1^−/−^ target genes differed among the four therapy groups. In some genes, the results even indicated loss of single cell clones (APC, Tmem60, and Casc3). (**C**) Representative band shifts of two cMS loci in Tmem60 and Senp6 target genes. Wt—wildtype; wt/−1—wildtype/−1 s.

The therapies not only induced immunological changes but also influenced the mutational frequency in Mlh1^−/−^ tumors (Figure 7). Both α-PD-L1 mono- and combinational therapy increased the overall mutation frequency of the tumor (Figure 7A). Figure 7B shows the mutation frequencies in dMMR-target genes. Frameshift mutations in APC, Tmem60, and Casc3 were no longer detectable upon treatment, likely because of eradicating single mutated cell clones. By contrast, novel mutations appeared (Figure 7B,C). Mutation frequencies in cMS repeats of MDC1 slightly increased after α-PD-L1 monotherapy and CTX. Additionally, cMS mutation frequencies in Senp6, Mbd6, and Lig4 increased after α-PD-L1 monotherapy. Since this trend was not seen in the combination, specific elimination is likely. Lig4 is the only exception. Here, cMS mutations were only detectable upon treatment but remained comparably low in the combination. CTX monotherapy alone triggered mutations in cMS of MDC1, Mbd6, and Lig4. 

## 3. Discussion

In the present study, we targeted the PD-1/PD-L1 axis additional to conventional CTX to enhance the survival of Mlh1^−/−^ mice. This strategy is based on the idea of utilizing CTX as an immunogenic cell death (ICD) inductor. Indeed, several reports from the literature provided sufficient data confirming ICD induction by cytostatic or cytotoxic drugs. These include, among others, anthracyclines such as doxorubicin, mitoxantrone, and oxaliplatin [23,24,25,26]. Besides, we recently described successful ICD induction by the deoxycytidine nucleoside analogue gemcitabine, characterized by reduced amounts of intracellular IDO-1, increased levels of surface-bound CalR, and elevated HMGB1 secretion [20]. To boost CTX-initiated immune responses, we here added an α-PD-L1 antibody and performed in vitro as well as in vivo analyses. Using a semi-autologous in vitro co-culture system, we provide evidence for the successful targeting of dMMR epithelial Mlh1^−/−^ murine tumor cells. However, the therapeutic outcome was highly individual among the two cell lines, A7450 T1 M1 and 328, nicely reflecting the heterogeneity among dMMR tumors patients, even in cases harboring the same MMR mutation [27,28]. The cell line Mlh1^−/−^ 328 is highly resistant and representative of a low-immunogenic subtype [21,22], which was confirmed further in this study. For the A7450 T1 M1 cells, the combination proved superior compared to either monotherapy with CTX or α-PD-L1 blockade. By studying the immune cells’ phenotype in-depth, we identified differences in specific activation and immune-regulatory markers. Numbers of PD-L1^+^ and PD-1^+^ as well as CD4^+^CD25^+^Foxp3^+^ regulatory T cells were exclusively lower in lymphocytes co-cultured with A7450 T1 M1 cells and the combination therapies. In 328 cells, no such differences were seen. Successful elimination of tumor cells is thus a likely result of creating an ICD-mediated inflamed, immunogenic tumor environment that enabled T cell-mediated killing. However, this regimen may only succeed in cases of at least moderate tumor immunity.

Using the Mlh1^−/−^ mouse model, we transferred the therapeutic CTX-α-PD-L1 therapy approach in the preclinical situation. Monotherapy of CTX or α-PD-L1 prolonged the overall survival of tumor-bearing mice, which was additionally improved by applying the combination of both agents. Hence, this setting proved safe and effective in our hands. By applying PET/CT staging, induction of SD or PR was seen in >80% of mice, respectively, and thus confirmed successful long-term effects of the applied regimen. Monotherapy of either agent yielded objective response in roughly 25% of mice, matching with clinical data, in which comparable or even superior response towards ICB compared to CTX monotherapy was already proven [29,30]. By combining immunogenic CTX, outcomes for patients with solid tumors were considerably improved. An example of successful chemo-immunotherapy is the combination of platinum CTX with PD-1/PD-L1 inhibitors [31,32,33], which has now become the standard 1st-line option for advanced PD-L1^+^ non-small cell lung cancer [34].

With the aim of inducing long-term immunological memory, longitudinal immune profiling revealed elevated plasma levels of IL13, TNFα, EOTAXIN, and MIP1β in the combination, accompanied by higher numbers of cytokine-secreting T-helper cells. The latter is likely attributable to the α-PD-L1 antibody since MIP1β levels additionally increased under this monotherapy. CTX monotherapy did not influence cytokines, with levels being comparable to controls. Another study on pancreatic carcinomas described the consistently high synthesis of CCL/CXCL chemokines and TGF-β-associated signals by gemcitabine after long-term treatment [35]. Alteration of disease course after combined gemcitabine and α-PD-1 treatment was only reached if mice also underwent genetic or pharmacologic ablation of TGF-β signaling. Though we have not analyzed TGF-β-signaling in depth, one may speculate that if induced at all, the α-PD-L1 antibody itself may have neutralized such effects. In support of this, we detected higher numbers of circulating CD83^+^ cells as well as CD3^+^CD8^+^ cytotoxic T cells in the combination group. Both cell types are involved in immune-mediated inflammation, either as antigen-presenting or effector cells. Hence, the dosing interval seems pivotal. CTX was given here three times with long treatment-free intervals, rendering the establishment of additional immunosuppressive cytokine barriers quite unlikely. Another point worth mentioning is the missing impact on circulating MDSCs. This highly immunosuppressive myeloid subtype is well-known for its capacity to facilitate tumor progression—both directly and indirectly [36,37,38,39,40]. The notion that we did not see any differences in numbers of circulating and splenic MDSCs in any treatment group matches with recent studies [41] and may explain the final relapse of Mlh1^−/−^ tumors. Generally, a high frequency of memory cells and low numbers of immunosuppressive cells are indicative of a good response, though individual differences exist [42]. The Mlh1^−/−^ mouse model is representative for immunosuppressive subtypes, thus, converting immune regulatory cells into pro-inflammatory cell types seem challenging. Still, the combination was able to shape the local tumor microenvironment. This included effective elimination of MDSCs and massive reduction of M2 macrophages even after several weeks of after-care, and vice versa; cytotoxic T lymphocytes increased, attributable to successful re-activation of antigen-driven immune responses. This finding is in line with increasing evidence on a broader remodeling of the tumor microenvironment by ICI than previously anticipated [43]. Although the lymphoid compartment is the main target for ICI, other immune cell populations, including myeloid cells, are affected as well [44]. In support of this, we also found higher levels of IRF5^+^ cells within tumor sections treated with the α-PD-L1 antibody alone or in the combination, often co-localized with PD1^+^ cells. IRF5 regulates type I IFN signaling and cytokines/chemokines with lymphocyte-chemotactic activities, such as RANTES. MIP1α/β and is considered a specific marker of inflammatory macrophages [45]. Such positive immune-modulating effects were mainly attributable to the α-PD-L1 antibody, while the CTX itself shaped the tumor microenvironment less efficiently. Here, numbers of MDSCs remained similar between controls and treated mice. Another recent study even reported boosted intratumoral MDSC accumulation by 5-FU that counteracted T and NK cell infiltration, thus abrogating the anti-tumor efficacy of PD-L1 blockade [46]. Such immunosuppressive effects were not seen in our study. In contrast, a positive effect on the immune system was seen here, by slightly elevated numbers of circulating as well as tumor-infiltrating cytotoxic and T helper cells. Other studies likewise reported very low toxicity on T cells and increased CD8^+^ cytotoxic T cell infiltration upon combined application of low, non-cytotoxic doses of gemcitabine, a Chk1 Inhibitor, and **α**-PD-L1 antibody [19,47]. Hence, gemcitabine is indeed a very interesting and promising backbone for combination therapies with ICIs. 

While dMMR constitutes a predictive biomarker for ICI-based regimens, recent evidence demonstrates that AT-rich interaction domain 1A (ARID1A) deficiency is associated with high antitumor immunity and good response towards ICI-monotherapy [48]. Indeed, tumors arising in Mlh1^−/−^ mice harbor multiple *ARID1A* missense mutations, resulting in loss-of-function of this tumor suppressor [22]. Hence, the observed clinical response seen here upon mono- and combination therapy with α-PD-L1 adds another piece of evidence for the causative relevance of *ARID1A* mutations in dMMR-driven cancers. In support of this, a complete pathologic response after two months of combined mFOLFOX6 with pembrolizumab therapy was recently reported in a Lynch syndrome patient suffering from an ARID1A mutated and tumor mutational burden (TMB) high dMMR CRC [49]. It is therefore tempting to speculate that the combination therapy is indeed beneficial for *ARID1A*-mutated dMMR tumors. Besides, the finding that PD-L1 expression is generally low in dMMR CRCs and not predictive in response to ICIs [50,51] warrants further investigations on *ARID1A* mutation status as a predictive biomarker. 

Another interesting finding of our current study was the observed striking difference in the mutational profile of the selected cMS marker. The monotherapies as well as their combination altered cMS frequency. Single mutated clones vanished, especially in the combination. By contrast, novel mutations appeared under CTX or ICI monotherapy and provide another explanation for final relapse. Though not analyzed in detail here, we speculate higher TMB after mono- than combination therapy. Notably, the most significant changes were seen after α-PD-L1 monotherapy.

Finally, the patients’ responses towards ICB are so individual, and determinants of such distinct reactions are just at the beginning of being understood. There is an increasing body of evidence pointing towards TMB, immune cell densities, and types in the tumor microenvironment, as well as expression levels of PD-1/PD-L1 and cytokines as legitimate factors. Elucidating the determinants of response and resistance are key to improving treatment strategies prospectively. 

## 4. Materials and Methods

### 4.1. Cell Culture

Mlh1^−/−^ tumor cells were established in our lab and basically characterized [22,52]. Cells were cultured in DMEM medium, supplemented with 10% FCS (fetal calf serum), 6mM Glutamine, and antibiotics (all from Biochrom, Berlin, Germany). Prior to analysis, cells were harvested, washed with PBS, and counted.

### 4.2. Colony Formation Assay

Cells were cultured as described above. For colony formation assay, a standard protocol was used as described before [53]. Briefly, 500 cells per well were seeded in a 6 well plate and incubated overnight. Thereafter, cells were treated with 0.24 nM gemcitabine or left untreated. After 6 days medium was removed and remaining cells were stained with 500 μL 0.2% crystal violet for 10 min on a rocking plate. Then, the wells were washed 5 times with PBS. For the second group, drug-containing medium was removed after 6 days, and cells were rested with medium for additional 6 days. Afterwards, the amount of colonies was analyzed using ImageJ-win64. 

### 4.3. Co-Culture Experiments

Harvested cells were stained with 5 μM CMFDA for 15 min at 37 °C. Cells were washed with PBS and seeded in a 24 well plate at a density of 20,000 cells per well. On the next day, 0.24 nM gemcitabine was added. Immune cells were harvested from peripheral blood samples routinely taken from Mlh1^−/−^ mice. Around 100 µL of pooled blood was incubated with erythrocyte lysis buffer (155 mM NH4Cl (MERCK Millipore, Darmstadt, Germany), 10 mM KHCO3 (MERCK Millipore), and 0.1 mM EDTA (Applichem, Darmstadt, Germany)) for 15 min, then stopped with PBS. Approximately 200,000 blood cells (E:T ratio: 1:10) were seeded per well. After 24 h, α-PD-L1 (10 μg/mL) was added. Tumor cells were counted with fluorescent microsphere beads (1.4 × 105 beads/mL, size: 10 µm, Polysciences, Hirschberg an der Bergstrasse, Germany) on a Flow Cytometer (BD FACSVerse™, BD Pharmingen, Heidelberg, Germany). Data analysis was performed using BD FACSuite software (BD Pharmingen). Additionally, immune cells were stained with a panel of conjugated monoclonal antibodies (mAb, 0.125 μg to 1.5 μg each) and measured on a spectral flow cytometer (Cytek™ Aurora, Amsterdam, The Netherlands). Data were analyzed using SpectroFlow™ Version 2.2.0.3.

### 4.4. Mlh1^−/−^ Mouse Model and In Vivo Treatment Protocol

#### 4.4.1. Institutional Review Board Statement

The German local authority approved all animal experiments on 27 June 2017: “Landesamt für Landwirtschaft, Lebensmittelsicherheit und Fischerei Mecklenburg-Vorpommern” (approval number: 7221.3-1-026/17; -026/17-3), under the German animal protection law and the EU Guideline 2010/63/EU. Mice were bred in the animal facility of the University Medical Center in Rostock under specific pathogen-free conditions. Mlh1 genotyping was done according to [21]. During their whole life-time, all animals got enrichment in the form of mouse-igloos (ANT Tierhaltungsbedarf, Buxtehude, Germany), nesting material (shredded tissue paper, Verbandmittel GmbH, Frankenberg, Germany), paper roles (75 × 38 mm, H 0528–151, ssniff-Spezialdiäten GmbH, Cologne, Germany), and wooden sticks (40 × 16 × 10 mm, Abedd, Vienna, Austria). During the experiment, mice were kept in type III cages (Zoonlab GmbH, Castrop-Rauxel, Germany) at 12-h dark:light cycle, the temperature of 21 ± 2 °C, and relative humidity of 60 ± 20% with food (pellets, 10 mm, ssniff-Spezialdiäten GmbH, Soest, Germany) and tap water ad libitum. 

#### 4.4.2. Experimental Protocol

Mice with PET/CT proven GIT (located in the duodenum) were taken into therapy. α-PD-L1 antibody (clone 6E11, kindly provided by Genentech, a subsidiary of Roche, South San Francisco, CA, USA) was dissolved in PBS and given intravenously (dose: 2.5 mg/kg bw, n = 10 mice) every 2 weeks, and gemcitabine (CTX, 100 mg/kg bw) intraperitoneal every 4 weeks (n = 10 mice), for a total of 3 times. In combination (n = 9 mice), CTX was given first, and after four weeks, α-PD-L1 antibody treatment started. Control mice were untreated (n = 9 mice). A treatment schedule is depicted in Figure 1. To prevent suffering of the mice, soaked pellets were offered and, additionally, humane endpoints (weight loss >15%, pain/distress, changes in social behavior) were applied. Before mice became moribund, they were sacrificed and blood, spleen, lymph nodes and GIT were removed for further analyses.

### 4.5. PET/CT Imaging

PET/CT imaging scans were performed on a small animal PET/CT scanner (Inveon PET/CT, Siemens Medical Solutions, Knoxville, TN, USA) according to a standard protocol as described before [54,55]. The PET image reconstruction method consisted of a 2-dimensional ordered subset expectation maximization algorithm (2D-OSEM) with four iterations and six subsets. Attenuation correction was performed on the basis that whole-body CT scan and a decay correction for [18F] was applied. PET images were corrected for random coincidences, dead time, and scatter. By marking the entire tumors, starting at the edge and cutting through the whole ^[18F]^FDG-enriched tumor, volumes and SUVs were determined using Inveon Research Workplace 4.2 software (Siemens Medical Solutions USA, Knoxville, Tennessee).

### 4.6. Immune Phenotyping

Blood samples were taken from anaesthetized mice every 6 weeks (retrobulbar venous plexus). Single cell suspensions of spleens and GIT were obtained upon passing them through a cell strainer (100 µm). Samples (2 × 10^5^/well) were stained with a panel of conjugated monoclonal antibodies (mAb, 1 μg each) followed by lysis of erythrocytes. Negative controls consisted of lymphocytes stained with the appropriate isotypes (Biolegend, San Diego, CA, USA) or unstained cells. Cells were washed, solved in PBS, and analyzed on a flow cytometer (BD FACSVerse™, BD Pharmingen). Data analysis was performed using BD FACSuite software (BD Pharmingen).

### 4.7. Procartaplex Cytokine Assay

Cytokine levels in plasma samples were determined according to the manufacturer’s instructions of the Procartaplex™ multiplex immunoassay (Thermo Fisher Scientific, Schwerte, Germany). Measurement and cytokine quantification was performed on a Bioplex 2000 (Bio-Rad Laboratories GmbH, Munich, Germany) in combination with the Bio-Plex Manager Software. Absolute plasma cytokine and chemokine level are presented [pg/mL].

### 4.8. Fragment Length Analysis of cMS Target Genes

Fragment length analysis was done from multiplexed PCRs of gDNA (25 ng/sample) from tumor and normal tissue as described [22]. To identify potential Mlh1 target genes, a panel (n = 20 marker) was screened. Primers were designed using Primer3 software (Elixir Estonia, Tartu, Estonia) to yield short amplicons (≤200 bp). Frameshift mutations were detected by mono- and/or biallelic band shifts, usually characterized by deletions. 

### 4.9. Immunofluorescence

Air-dried cryostat sections (4 μm thickness) were fixed (methanol, 8 min). Unspecific binding sites were blocked in 2% BSA (Roth, Karlsruhe, Germany) for 1 h followed by incubation with 1 μg of the following AlexaFluor488, AlexaFluor594, and AlexaFluor 647-labeled mAbs: CD4, CD8α, CD11b, Gr1, CD11c, F4/80, CD104, CD206, PD-1, and PD-L1 (all from Biolegend). For intracellular stainings, slides were fixed in 4% paraformaldehyde w/o methanol (Thermo Scientific, Darmstadt, Germany, 30min) and cells permeabilized in 0.5% Triton X−100 (Sigma-Aldrich, Darmstadt, Germany, 15 min). After blocking with 2% BSA (Serva, Heidelberg, Germany), slides were incubated with the monoclonal rabbit anti-IRF5 antibody (1:50; ThermoFisher Scientific, Darmstadt, Germany) overnight at 4 °C, followed by a secondary goat anti-rabbit Alexa647 antibody (1:500; Cell Signaling, Frankfurt am Main, Germany). Sections were washed, embedded in Roti Mount Flour Care DAPI (Roth), and target proteins visualized on a confocal laser scanning microscope (Elyra 7, Zeiss, Jena, Germany) using 20× objectives. IRF5^+^ cells were quantified by counting individual positive cells in three high power fields per sample (n = 3/group).

### 4.10. Statistics

All values are expressed as mean ± SD. After proving the assumption of normality (Kolmogorov-Smirnov test), differences between vaccinated and control mice were determined using the unpaired Student’s *t*-test or one-way ANOVA (Bonferroni or Dunnett’s multiple comparison). Kaplan-Meier survival analysis was done by applying the log rank (Mantel Cox) test. Statistical analyses were performed using GraphPad Prism 8.0.2 (San Diego, CA, USA). The criterion for significance was set to *p* < 0.05.

## 5. Conclusions

The combination of gemcitabine and α-PD-L1 prolongs the lifetime of Mlh1^−/−^ mice significantly via long-term tumor growth control. The treatment modulates the tumor microenvironment by eliminating MDSCs and massive reductions of M2 macrophages counterbalanced by an increase in cytotoxic T cells. Patients would profit from a combinational chemo- and ICI therapy, to prevent development of resistance mechanisms.

## Data Availability

Data are available from the corresponding author upon reasonable request.
